# Tracing the evolutionary trajectory of the IncP-2 plasmid co-harboring *bla*_IMP-45_ and *bla*_VIM-1_: an outbreak of *Pseudomonas aeruginosa* co-producing IMP-45 and VIM-1 carbapenemases in China

**DOI:** 10.3389/fcimb.2025.1623241

**Published:** 2025-06-19

**Authors:** Yiqun Ma, Zichen Lei, Yulin Zhang, Qi Liu, Feilong Zhang, Hao Zu, Xinrui Yang, Ziyao Li, Binghuai Lu

**Affiliations:** ^1^ Peking University China-Japan Friendship School of Clinical Medicine, China-Japan Friendship Hospital, Beijing, China; ^2^ Laboratory of Clinical Microbiology and Infectious Diseases, Department of Pulmonary and Critical Care Medicine, Beijing Key Laboratory of Surveillance, Early Warning and Pathogen Research on Emerging Infectious Diseases, National Center for Respiratory Medicine, China-Japan Friendship Hospital, Beijing, China; ^3^ Institute of Respiratory Medicine, Chinese Academy of Medical Sciences, Beijing, China; ^4^ China-Japan Friendship Institute of Clinical Medical Sciences, China-Japan Friendship Hospital, Beijing, China; ^5^ Peking Union Medical College, Chinese Academy of Medical Sciences, Beijing, China; ^6^ Yanjing Medical College, Capital Medical University, Beijing, China

**Keywords:** carbapenem-resistant *Pseudomonas aeruginosa*, CRPA, horizontal gene transfer (HGT), nosocomial infection, metallo-beta-lactamases (MBL), resistance dissemination

## Abstract

**Background:**

Carbapenem-resistant *Pseudomonas aeruginosa* (CRPA) poses a significant global health risk, particularly for immunocompromised individuals. This study documents an outbreak of CRPA strains co-harboring *bla*_VIM-1_ and *bla*_IMP-45_ on IncP-2 plasmids in a Chinese tertiary hospital, resulting in poor outcomes for transplant patients.

**Methods:**

17 ST313 VIM-1-IMP-45 CRPA strains were collected from transplant patients, and antibiotic susceptibility was tested via microbroth dilution. Whole genome sequencing (WGS) identified drug resistance and virulence mechanisms, analyzed ST313 *P. aeruginosa* phylogeny, and traced *bla*_VIM-1_ and *bla*_IMP-45_ origins. Conjugation experiments were conducted to assess the conjugative potential of the IncP-2 plasmid co-harboring *bla*_VIM-1_ and *bla*_IMP-45_. Structural and molecular docking studies explored the PBP3 (P527S) mutation’s role in aztreonam resistance.

**Results:**

From February 2022 to July 2024, 17 ST313 VIM-1-IMP-45 CRPA strains from 10 transplant patients were identified. All strains were extensively drug-resistant but sensitive to colistin and cefiderocol. WGS showed *bla*_IMP-45_ and *bla*_VIM-1_ on an IncP-2 megaplasmid. Phylogenetic analysis indicated high homology with plasmids carrying *bla*_IMP-45_. Further analysis of the genetic environment showed that the IncP-2 plasmid co-harboring *bla*_VIM-1_ and *bla*_IMP-45_ was formed by the insertion of a Tn*3*-family transposon carrying *bla*_VIM-1_ into the IncP-2 plasmid carrying *bla*_IMP-45_. In addition aztreonam-resistant strains (14/15) had a PBP3 (P527S) mutation, with molecular docking studies suggesting reduced aztreonam binding.

**Conclusions:**

This study reports a clonal outbreak of ST313 *P. aeruginosa* strains co-producing IMP-45 and VIM-1 carbapenemases in a tertiary hospital. The evolutionary path of the IncP-2 plasmid co-harboring *bla*_IMP-45_ and *bla*_VIM-1_ was elucidated.

## Introduction

Carbapenem-resistant *Pseudomonas aeruginosa* (CRPA) is a major global public health threat, significantly increasing morbidity and mortality rates, especially among immunocompromised individuals (Azam and Khan, 2019). The treatment of CRPA infections is challenging due to its remarkable ability to resist multiple antibiotics (Pang et al., 2019). One of the mechanisms for carbapenem resistance is the production of metallo-β-lactamases (MBLs), mainly consisting of Verona integron-encoded metallo-β-lactamase (VIM) and imipenemase (IMP) (Cornaglia et al., 2011). Typically, VIM-1 is commonly found in *Enterobacteriaceae (* Walsh et al., 2005; Vatopoulos, 2008). Additionally, *P. aeruginosa* isolates with VIM-1 have been identified in Spain and Turkey (Malkoçoğlu et al., 2017; PÉrez-VÁzquez et al., 2020), but are rarely reported in China (Hong et al., 2015). Currently, only a few sporadic studies of VIM-1-producing *P. aeruginosa* have been reported in China (Kong et al., 2015; Cai et al., 2016; Jing et al., 2018; Kang et al., 2023).

In China, the presence of MBL genes such as *bla*_IMP-1_, *bla*_IMP-4_, *bla*_IMP-6_, *bla*_IMP-8_, *bla*_IMP-9_, *bla*_IMP-10_, and *bla*_IMP-45_ *(* Hong et al., 2015) has been documented. Initially, *bla*_IMP-9_ was detected in *P. aeruginosa* isolates from Guangzhou (Xiong et al., 2006), with subsequent outbreaks occurring in 2000 and between 2005 and 2007, respectively (Xiong et al., 2006; Chao et al., 2008). IMP-45, distinguished by a single amino acid substitution (G214S) in IMP-9, was first reported in 2014 in a *P. aeruginosa* strain of canine originated from Beijing, demonstrating increased resistance to meropenem compared to imipenem (Wang et al., 2014).

In this study, we report an outbreak of CRPA strains co-harboring *bla*_VIM-1_ and *bla*_IMP-45_ on IncP-2 plasmids in a tertiary hospital in China, leading to poor prognosis in transplant patients.

## Material and methods

### Bacterial isolation and clinical data collection and antimicrobial susceptibility testing

During February 2022 to July 2024, 17 nonduplicated CRPA strains co-harboring *bla*_VIM-1_ and *bla*_IMP-45_, collected from 10 patients, were enrolled in the study. Demographics and relevant clinical data of the patients were obtained through review of medical records. Susceptibility to ticarcillin-clavulanic acid, ceftazidime, cefoperazone-sulbactam, cefepime, meropenem, imipenem, amikacin, tobramycin, ciprofloxacin, levofloxacin, and colistin were measured using VITEK^®^2 system (BioMérieux, Marcy l’Étoile, France) following the manufacturer’s instructions. Minimal inhibitory concentrations (MICs) were determined for cefiderocol, piperacillin-tazobactam, aztreonam (AZT) and ceftazidime/avibactam (CZA) by broth microdilution method and interpretation was according to recommendations of the CLSI (Lewis and James, 2022), *P. aeruginosa* ATCC27853 served as quality control strains.

### Comparative genomic analysis of *bla*_IMP-45_ carrying plasmids

To better understand the features of genetic environments surrounding *bla*_IMP-45_ and *bla*_VIM-1_ genes, we searched the Genbank database on NCBI and obtained 15 intact plasmids harboring *bla*_IMP-45_ worldwide from China as of October 1, 2024.

### Phylogenetic analysis

As of October 1, 2024, 53 P*. aeruginosa* genomes assigned to ST313 were downloaded from the RefSeq database on NCBI, We excluded strains with unknown country of origin and collection time, retaining 46 ST313 *P. aeruginosa* isolates and 17 ST313 VIM-1-IMP-45 CRPA strains for phylogenetic analysis. The phylogenetic tree for all ST313 *P. aeruginosa* genomes was constructed according to their SNPs by using Snippy version 4.6.0 (https://github.com/tseemann/snippy). ST313 *P. aeruginosa* genomes’ SNPs were filtered to remove recombination using Gubbins v3.3.5 (Croucher et al., 2015). The phylogenetic tree of the IncP-2 plasmids carrying blaIMP-45 and the IncP-2 plasmids co-harboring *bla*_VIM-1_ and *bla*_IMP-45_ were also constructed based on their SNPs using Snippy version 4.6.0. All maximum likelihood phylogenetic trees were inferred from a core genome alignment by using Raxml v8.2.12 (Stamatakis, 2014). The specific parameters for Snippy were: alignment coverage threshold –minfrac = 0.9 and minimum depth –mincov = 10. For Gubbins, we used maximum iterations = 5 and P-value threshold = 0.05. All trees were visualized in iTOL (https://itol.embl.de).

### Cefiderocol induction resistance experiments and conjugation assay of plasmid co-carrying *bla*_VIM-1_ and *bla*_IMP-45_

To induce cefiderocol resistance in *P. aeruginosa*, we followed a previously reported method (Nurjadi et al., 2022) with slightly modifications. Briefly, The amikacin-sensitive clinical single colony of *P. aeruginosa* AS01 strain grown on Columbia agar supplemented with 5% sheep blood was inoculated into 5 mL of cation-adjusted Mueller-Hinton broth (CA-MHB) (BD Diagnostics, Germany) and incubated at 37°C with constant shaking at 200 rpm for 18 hours. After overnight incubation, 100 μL of the culture was transferred into a fresh 5 mL of CA-MHB, and cefiderocol (Shionogi, Japan) was added to achieve a final concentration of 0.5 mg/L. The culture was then incubated under the same conditions as the initial culture. This process was repeated daily, with the cefiderocol concentration doubled at each passage, until no visible turbidity or bacterial growth was observed after overnight incubation, or until the cefiderocol concentration reached 64 mg/L. For population analysis profiling, 10 μL of the bacterial suspension from the overnight culture was plated onto Columbia blood agar, and 10 single colonies were randomly selected for cefiderocol susceptibility testing using broth microdilution. Conjugation experiments were performed with strain AS02, which exhibited resistance to cefiderocol but remained susceptible to amikacin and was selected as the recipient strain, while strain CJ05 served as the donor strain. Donor and recipient bacteria were separately cultured in LB broth at 37°C until reaching logarithmic growth phase (OD600 = 0.6-0.8), then mixed in a 1:1 ratio and incubated at 37°C overnight for conjugation. The conjugation mixture was subjected to 10-fold serial dilution and plated on Mueller-Hinton agar containing cefiderocol (4 mg/L) and amikacin (16 mg/L), followed by incubation at 37°C for 18–24 hours. Putative transconjugants were confirmed by whole-genome sequencing. Additional conjugation experiments were conducted using *Escherichia coli* J53 as an alternative recipient strain. Each conjugation experiment was repeated three times. The antimicrobial susceptibility tests mentioned above were performed on strains AS01, AS02, and transconjugant TC01 using the VITEK^®^2 system and broth microdilution.

### Whole-genome sequencing and bioinformatics analysis

Genomic DNA from VIM-1-IMP-45-CRPA strains CJ01-CJ17, and transconjugant strain TC01 was extracted and were sequenced using an Illumina NovaSeq PE150 at the Beijing Novogene Bioinformatics Technology Co., Ltd. Raw reads were filtered to remove low-quality sequences and adaptors. *De novo* assembly was conducted using SOAP *de novo* 2.04 (Li et al., 2008; Li et al., 2010), SPAdes 3.10.0 (Bankevich et al., 2012), and ABySS 1.3.7 (Simpson et al., 2009). The assembly results were integrated with CISA 1.3 software. The gap in preliminary assembly results was filled using Gapcloser 1.12. In addition, whole-genome sequences for CJ05, AS01 and AS02 were obtained through Illumina NovaSeq PE150 and nanopore sequencing on MinION flow cells. Hybrid assemblies of Illumina short reads and MinION long reads were prepared with Unicycler version 0.4.8 (Wick et al., 2017). Multilocus sequence typing (MLST) was performed by using an MLST tool (https://github.com/tseemann/mlst). Antimicrobial drug resistance and virulence genes were identified with ABRicate version 1.0.0 (https://github.com/tseemann/abricate) according to the National Center for Biotechnology Information (NCBI) AMRFinderPlus (https://www.ncbi.nlm.nih.gov) and virulence factor (http://www.mgc.ac.cn/VFs) databases. Pairwise single-nucleotide polymorphism (SNP) distance was evaluated with Snp-dists (https://github.com/tseemann/snp-dists). Sequence comparisons were performed by using Easyfig version 2.2.3 and annotated by Prokka and Bakta (Seemann, 2014; Schwengers et al., 2021). Alignments and visualization of plasmids were generated by BLAST Ring Image Generator (BRIG) software version 0.95 (https://sourceforge.net/projects/brig). Linear alignments of bla_IMP-45_-bearing structures were generated using ggplot2 and gggenes in R-4.4.2. The heatmap was translated using TBtools-II (Chen et al., 2023). Platon (Schwengers et al., 2020) was used to predict plasmids in transconjugant TC01 and all ST313 VIM-1-IMP-45-CRPA except CJ05.

### Structure modeling and molecular docking

Retrieving the RCSB Protein Data Bank (PDB), the crystal structures of PBP3 complexed with aztreonam (PDB DOI: https://doi.org/10.2210/pdb3PBS/pdb) was applied as the structure template in the present study (Han et al., 2010). After PSI-BLAST (Altschul et al., 1997) and MUSCLE alignment (Edgar, 2022), the homology models of PBP3(P527S) were both built in Schrödinger. The protein structure was refined and preprocessed using the OPLS4 force field (Lu et al., 2021), following the ligands preparing for diverse ionization states and isomers. In the covalent docking, the aztreonam of PBP3 complexed with aztreonam was set as the centroid of grid box, and the β-lactam was chosen as reaction type for pose prediction. After completion of docking, the Prime MM-GBSA scores of the top one output poses for each ligand was calculated for comparing the binding affinities of the ligands (Li et al., 2011).

## Result

### Outbreak of ST313 *P. aeruginosa* co-harboring *bla*_IMP-45_ and *bla*_VIM-1_

**Figure 1:**
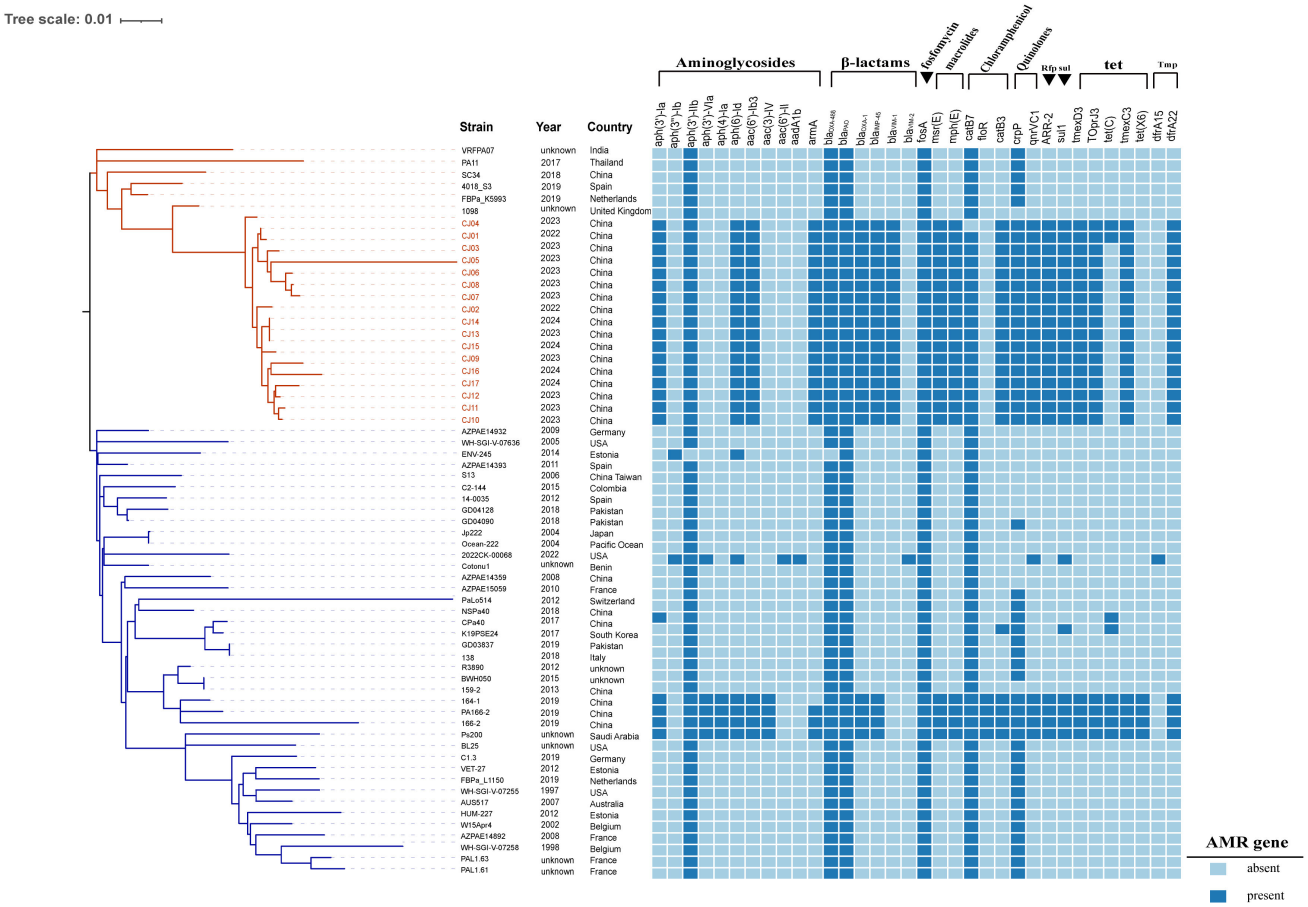
Phylogenetic analysis and epidemic distribution of ST313 *P. aeruginosa*. 63 ST313 *P. aeruginosa* phylogenetic analysis, *bla*_VIM-1_ and *bla*_IMP-45_ resistance genes and strains carrying *bla*_VIM-1_ and *bla*_IMP-45_ genes are marked in red, AMR gene: Antimicrobial resistance gene. In the strains, red markings indicate VIM-1–IMP-45 CRPA strain. In the phylogenetic tree, the blue branches represent subgroup1, and the red branches represent subgroup2.

During 2022-2024, 17 ST313 *P. aeruginosa* strains co-harboring *bla*_IMP-45_ and *bla*_VIM-1_ were identified, including 2 isolates from 2022, 11 from 2023, and 4 from 2024. Following comprehensive disinfection measures implemented in the ward, no further occurrences of VIM-1-IMP-45-CRPA strains were detected. The VIM-1-IMP-45-CRPA strains were isolated from 10 immunocompromised patients, all of whom had undergone lung or kidney transplants (**Table 1**). The cohort consisted of 9 males and 1 female, aged between 40 and 70 years, all of whom presented with complex medical histories. Among the 10 patients, 8 had VIM-1-IMP-45-CRPA detected in their bronchoalveolar lavage fluid (BALF), of whom 7 received ceftazidime/avibactam combined with aztreonam (CZA+AZT) treatment. Another 2 patients had VIM-1-IMP-45-CRPA detected in their blood but did not receive CZA+AZT treatment. Among patients who received CZA+AZT treatment, the mortality rate was 12.5% (1/8), while among patients who did not receive CZA+AZT treatment, the mortality rate was 100% (3/3), with 66.7% (2/3) of the deaths associated with bloodstream infections (**Table 1**). Through phylogenetic analysis of 63 ST313 genomes, (46 from the NCBI Reference Sequence database and 17 from this study), using the midpoint rooting method, we found that ST313 *P. aeruginosa* can be divided into two subgroups (**Figure 1**), revealing contrasting evolutionary trajectories and transmission dynamics. Subgroup 2 demonstrated tight clonal clustering with multiple closely related strains predominantly originating from China during 2017-2024, indicating recent clonal expansion and suggesting a single-source outbreak with rapid person-to-person transmission capabilities. In contrast, subgroup 1 exhibited greater phylogenetic diversity with strains distributed across multiple countries and spanning a broader temporal range (1997-2022), reflecting longer evolutionary timescales and endemic circulation patterns. The transmission timeline analysis revealed two distinct epidemiological phases: a recent epidemic emergence in subgroup 2 concentrated within a 2-year period, and historical global circulation in subgroup 1 spanning over two decades. Notably, the evolutionary relationship analysis demonstrated that while Subgroup 1 maintained ancestral ecological flexibility with 27.5% (11/40) environmental or animal isolates (**Supplementary Table S3**) distributed throughout the phylogeny, subgroup 2 exhibited marked anthropocentric specialization with only a single animal isolate among an otherwise exclusively human-derived population.

**Table 1:** Clinical data of 10 patients infected by *Pseudomonas aeruginosa* co-producing VIM-1 and IMP-45 carbapenemases, 2022–2024^α^.

Patient ID	Strain	ST	Age(year)	Gender	Ward	Medical history	Specimen collection date	Antibiotic treatment	Specimen type	Outcome/date
1	CJ01	ST313	70	Male	LTD	Post-lung transplantation	2022.04.04	COL, CZA+AZT, AZT+COL	BALF	Death/2023.02.24
	CJ03	ST313					2023.02.18			
2	CJ02	ST313	66	Male	LTD	Post-lung transplantation	2022.10.09	MEM+LVX, FOS+COL	Blood	Death/2023.10.18
3	CJ04	ST313	55	Male	LTD	Post-lung transplantation	2023.04.20	CZA+AZT, TZP+AZT, AZT	BALF	Discharge/2024.06.25
4	CJ05	ST313	40	Female	LTD	Post-lung transplantation	2023.05.16	CZA+TZP	BALF	Death/2023.05.20
5	CJ06	ST313	61	Male	PCCM	Post-kindey transplantation	2023.06.20	CZA+AZT, TZP, SCF, CZA+COL	BALF	Discharge/2023.07.22
6	CJ07	ST313	31	Male	LTD	Post-lung transplantation	2023.07.07	MEM, CZA+AZT	BALF	Discharge/2023.07.19
7	CJ08	ST313	45	Male	LTD	Post-lung transplantation	2023.08.16	CZA+AZT, AZT, COL	BALF	Discharge/2024.07.08
8	CJ09	ST313	66	Male	LTD	Post-lung transplantation	2023.08.31	CZA+AZT, COL	BALF	Discharge/2024.09.30
	CJ13	ST313					2023.10.25			
	CJ15	ST313					2024.02.29			
9	CJ10	ST313	66	Male	LTD	Post-lung transplantation	2023.09.19	CZA, AZT+COL, CZA+AZT, TZP	BALF	Discharge/2024.08.23
	CJ11	ST313					2023.09.28			
	CJ12	ST313					2023.10.12			
	CJ14	ST313					2024.01.18			
10	CJ16	ST313	61	Male	LTD	Post-lung transplantation	2024.07.29	MEM	Blood	Death/2024.07.31
	CJ17	ST313								

^α^AZT, aztreonam; BALF, bronchoalveolar lavage fluid; COL, colistin; CZA, ceftazidime/avibactam; FOS, fosfomycin; IMP, imipenemase; LTD, lung transplantation department; LVX, levofloxacin; MEM, meropenem; PCCM, pulmonary and critical care medicine; SCF, sulbactam/cefoperazone; TZP, piperacillin/tazobactam; VIM, Verona integron-encoded metallo-β-lactamase.

### Characteristics of VIM-1-IMP-45-CRPA strains

All 17 VIM-1-IMP-45-CRPA strains isolates shared almost identical resistance genes, differing by 0–192 SNPs. Except for strain CJ05, the differences among the remaining strains range from 0 to 79 SNPs, demonstrating a high degree of relatedness among those isolates and clonal transmission of -producing *P. aeruginosa* ST313 in China (**Supplementary Figure S1**). Notably, all 17 ST313 VIM-1-IMP-45-CRPA strains possessed virulence genes similar to the highly virulent strain PA14, including the type III secretion system genotypes *exoU*+, *exoS*+ and *exoY*+(**Supplementary Figure S2**).

Drug susceptibility testing showed that, except for the CJ13 and CJ04, intermediate and sensitive to aztreonam, respectively, all ST313 VIM-1-IMP-45-CRPA strains were resistant to carbapenems (meropenem and imipenem), several β-lactams (ceftazidime, cefepime, piperacillin/tazobactam, cefoperazone/sulbactam, ceftazidime/avibactam, aztreonam), quinolones (ciprofloxacin, levofloxacin), and aminoglycosides (amikacin, tobramycin). But all strains remained sensitive to colistin and cefiderocol (**Table 2**).

**Table 2:** Antimicrobial drug susceptibility profiles for the 17 VIM-1–IMP-45–CRPA strains from China^*^.

Strains	MIC (μg/mL)														
	MEM	IMP	CAZ	TCC	FEP	AZT	AK	TOB	LEV	CIP	P/T	CZA	COL	SCF	FDC
CJ01	>16	>16	>64	>128	>32	>32	>64	>16	>8	>4	>128/4	>128/4	≤0.5	>64	0.5
CJ02	>16	>16	>64	>128	>32	>32	>64	>16	>8	>4	>128/4	>128/4	≤0.5	>64	0.5
CJ03	>16	>16	>64	>128	>32	>32	>64	>16	>8	>4	>128/4	>128/4	≤0.5	>64	0.5
CJ04	>16	>16	>64	>128	>32	2	>64	>16	>8	>4	>128/4	>128/4	≤0.5	>64	1
CJ05	>16	>16	>64	>128	>32	>32	>64	>16	>8	>4	>128/4	>128/4	≤0.5	>64	0.25
CJ06	>16	>16	>64	>128	>32	>32	>64	>16	>8	>4	>128/4	>128/4	≤0.5	>64	0.125
CJ07	>16	>16	>64	>128	>32	>32	>64	>16	>8	>4	>128/4	>128/4	≤0.5	>64	0.5
CJ08	>16	>16	>64	>128	>32	>32	>64	>16	>8	>4	>128/4	>128/4	≤0.5	>64	0.5
CJ09	>16	>16	>64	>128	>32	>32	>64	>16	>8	>4	>128/4	>128/4	≤0.5	>64	0.5
CJ10	>16	>16	>64	>128	>32	>32	>64	>16	>8	>4	>128/4	>128/4	≤0.5	>64	0.5
CJ11	>16	>16	>64	>128	>32	>32	>64	>16	>8	>4	>128/4	>128/4	≤0.5	>64	0.5
CJ12	>16	>16	>64	>128	>32	>32	>64	>16	>8	>4	>128/4	>128/4	≤0.5	>64	0.06
CJ13	>16	>16	>64	>128	>32	16	>64	>16	>8	>4	64/4	>128/4	≤0.5	>64	0.125
CJ14	>16	>16	>64	>128	>32	>32	>64	>16	>8	>4	>128/4	>128/4	≤0.5	>64	0.5
CJ15	>16	>16	>64	>128	>32	>32	>64	>16	>8	>4	>128/4	>128/4	≤0.5	>64	0.5
CJ16	>16	>16	>64	>128	>32	>32	>64	>16	>8	>4	>128/4	>128/4	≤0.5	>64	0.5
CJ17	>16	>16	>64	>128	>32	>32	>64	>16	>8	>4	>128/4	>128/4	≤0.5	>64	0.5
ATCC 27853	0.5	2	2	16	2	2	≤2	≤1	1	0.5	1/4	0.5/4	2	≤8	0.125

^*^ AK, amikacin; ATCC, American Type Culture Collection (https://www.atcc.org); AZT, aztreonam; CAZ, ceftazidime; CIP, ciprofloxacin; COL, colistin; CRPA, carbapenem-resistant *Pseudomonas aeruginosa*; CZA, ceftazidime/avibactam; FDC, cefiderocol; FEP, cefepime; IPM, imipenem; VIM, Verona integron-encoded metallo-β-lactamase; LEV, levofloxacin; MEM, meropenem; NA, not applicable; P/T, piperacillin/tazobactam; ST313, sequence type 313;SCF,cefoperazone/sulbactam; TOB, tobramycin; TCC, ticarcillin/clavulanic acid.

Interestingly, almost all aztreonam-resistant VIM-1-IMP-45-CRPA strains (14/15) harbored a mistranslation mutation (P527S) in PBP3. The non-mutated PBP3 protein was consistent with the PBP3 protein of the standard strain PAO1.Based on the PBP3(PAO1) structure in the RCSB PDB database, we used Schrödinger to predict the structure of the point-mutated PBP3 (P527S) to analyze interactions with aztreonam. Subsequently, molecular docking of aztreonam with both PBP3(PAO1) and PBP3(P527S) was performed using Schrodinger software (**Supplementary Figure S4**). The docking score of the PBP3(P527S) protein decreased (docking score: -9.911kcal/mol to -8.770kcal/mol). Subsequently the MM/GBSA binding free energy from the top docking pose of aztreonam to PBP3 was calculated. From our analysis, PBP3(P527S) exhibited less negative binding free energy values for aztreonam (-21.36 kcal/mol for PBP3(PAO1), -20.06 kcal/mol for PBP3(P527S)), suggesting the weakened binding affinity to PBP3(P527S).

### Evaluating the transferability of the IncP-2 plasmid co-harboring *bla*_VIM-1_ and *bla*_IMP-45_

To assess plasmid transferability, we selected strain CJ05 for further experiment. CJ05 strain contains an IncP-2 megaplasmid co-harboring *bla*_VIM-1_ and *bla*_IMP-45_ by whole-genome sequencing. Given all VIM-1-IMP-45-CRPA strains were resistance to amikacin but sensitive to cefiderocol, we chosen a clinical amikacin-susceptible *P. aeruginosa* strain AS01 for cefiderocol induction resistance experiments. An cefiderocol induced resistance *P. aeruginosa* strain, AS02, was used for conjugation experiments. The AS01 strain was identified as ST463 *P. aeruginosa*. The whole-genome sequencing revealed that it harbors a plasmid of 41,101 bp in size carrying the *bla*_KPC-2_ gene. Other resistance genes, including *aph(3’)-IIb*, *bla*_PAO_, *bla*_OXA-486_, *fosA*, *catB7*, and *crpP*, are located on the chromosome. Antibiotic susceptibility testing showed that the strain is resistant to carbapenems (meropenem and imipenem), several β-lactams (ceftazidime, cefepime, piperacillin/tazobactam, cefoperazone/sulbactam, ceftazidime/avibactam, aztreonam), and quinolones (ciprofloxacin, levofloxacin). However, it remains sensitive to aminoglycosides (amikacin, tobramycin), colistin, and cefiderocol (**Supplementary Table S2**). By analyzing the genome of the AS02 strain, we found that the *bla*_KPC-2_ gene on its plasmid had evolved into *bla*_KPC-33_, while other resistance genes remained identical to those in the AS01 strain. The antibiotic susceptibility profile of AS02 was similar to that of AS01, except that AS02 exhibited resistance to cefiderocol (**Supplementary Table S2**). Additionally, we observed mutations in certain genes in the AS02 strain, including *hasR*. Furthermore, conjugation experiments were conducted using *E. coli* J53 as the recipient strain. Conjugation experiments showed that the IncP-2 plasmid could be transferred within *P. aeruginosa*, but not to *E. coli* I53. Using Platon to analyze the plasmid sequence of the transconjugant TC01 revealed the presence of resistance genes, including *aac(6’)-Ib3*, *armA*, *bla*_KPC-33_, *bla*_IMP-45_ and *bla*_VIM-1_. Drug susceptibility test showed that it was resistant to amikacin and cefiderocol, but only sensitive to colistin (**Supplementary Table S2**).

### Evolutionary trajectory of the IncP-2 plasmid co-harboring *bla*_VIM-1_ and *bla*_IMP-45_

Using Platon predict plasmids in transconjugant TC01 and all ST313 VIM-1-IMP-45-CRPA strains except CJ05. Phylogenetic analysis of the system shows high homology in the IncP-2 plasmids of ST313 VIM-1-IMP-45-CRPA and transconjugant TC01 in the VIM-1-IMP-45-CRPA plasmids (**Supplementary Figure S3**).

**Figure 2:**
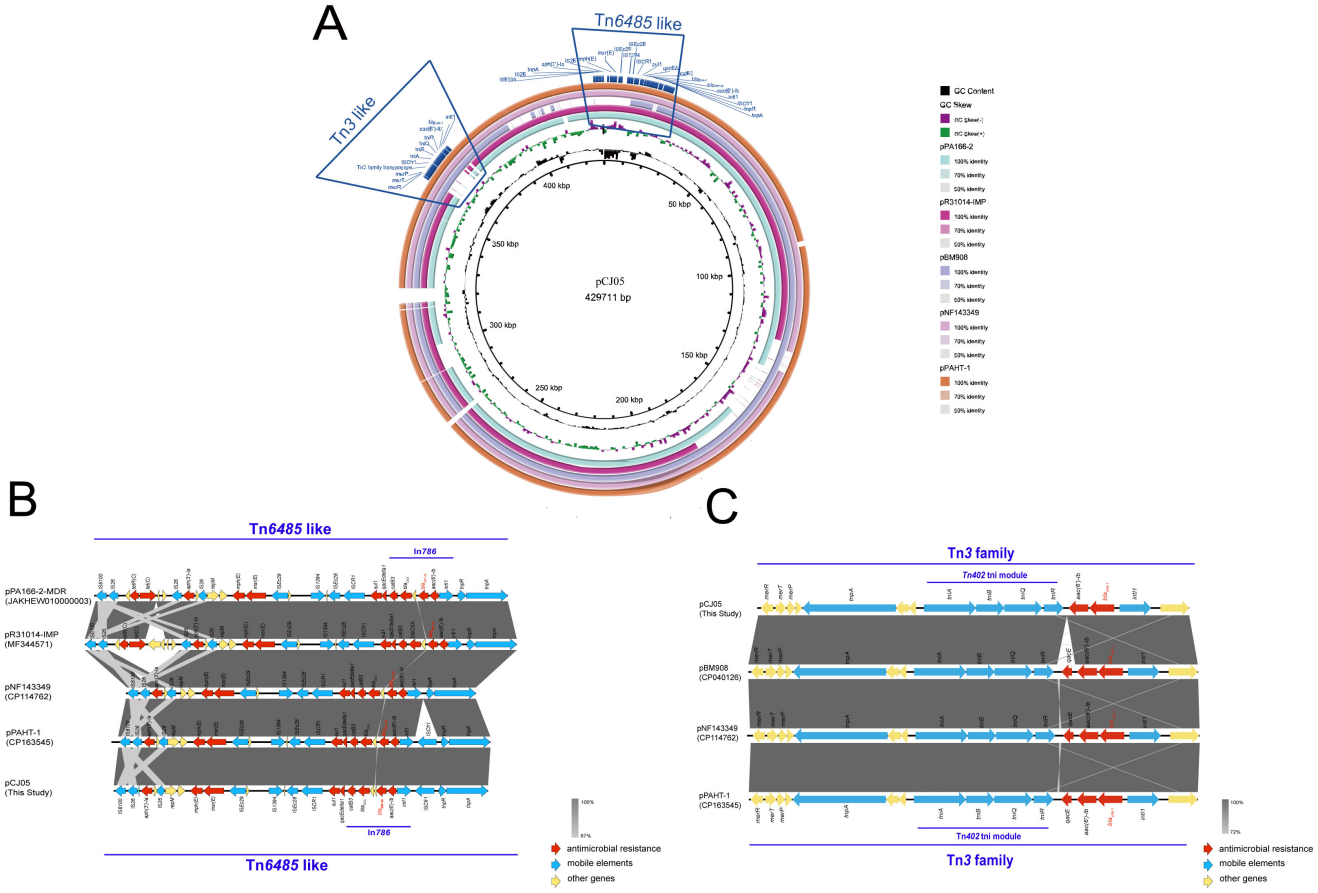
Genetic context of *bla*_IMP-45_ and *bla*_VIM-1_. **(A)** Genome comparison of plasmid pCJ05 co-harboring *bla*_VIM-1_ and *bla*_IMP-45_. Schematic map of plasmid pCJ05, this plasmid sequence was compared with plasmids pPA166-2(accession number:JAKHEW010000003), pR31014-IMP(accession number:MF344571), pNF143349(accession number:CP114762), and pPAHT-1(accession number:CP163545); **(B, C)** Linear characterization of Tn*6485-*like transposon carrying *bla*_IMP-45_ and Tn*3-*family transposon carrying *bla*_VIM-1_ in pCJ05 plasmid with similar sequence. Red, blue, and yellow arrows denote antimicrobial resistance genes, mobile elements, and other protein-encoding genes, respectively. The Δ symbol indicates that the gene is truncated.

**Figure 3:**
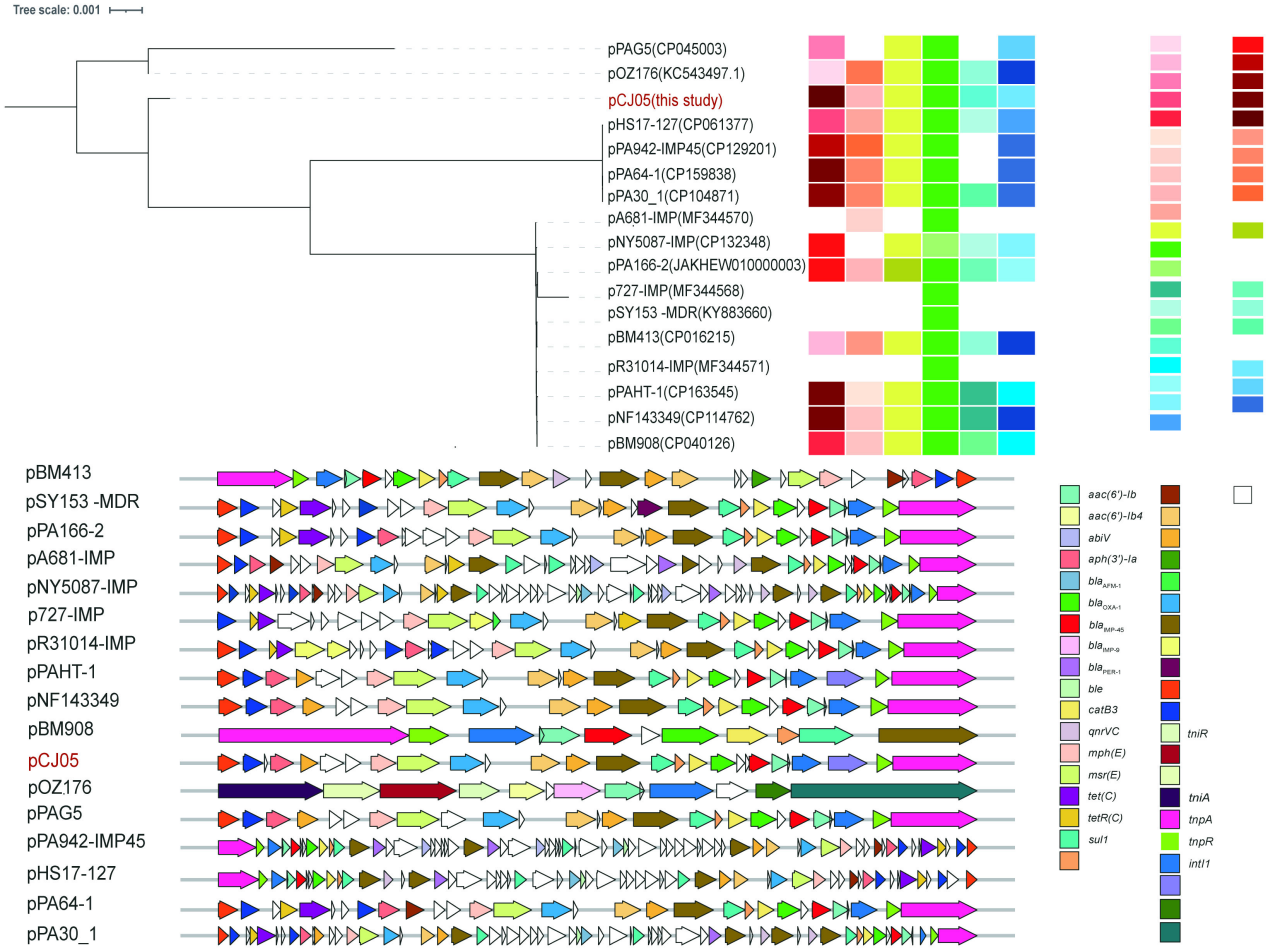
Evolution relationship and comprehensive information among IncP-2 plasmids carrying *bla*_IMP-45._ Plasmids were colored according to year, STs, host, strains, sample, country/province, and genetic context of *bla*IMP-45. The snp-based maximum likelihood phylogenetic tree was built by RaxML with pOZ176 as the reference. The Interactive Tree of Life (https://itol.embl.de) was used for visualization.

**Figure 4:**
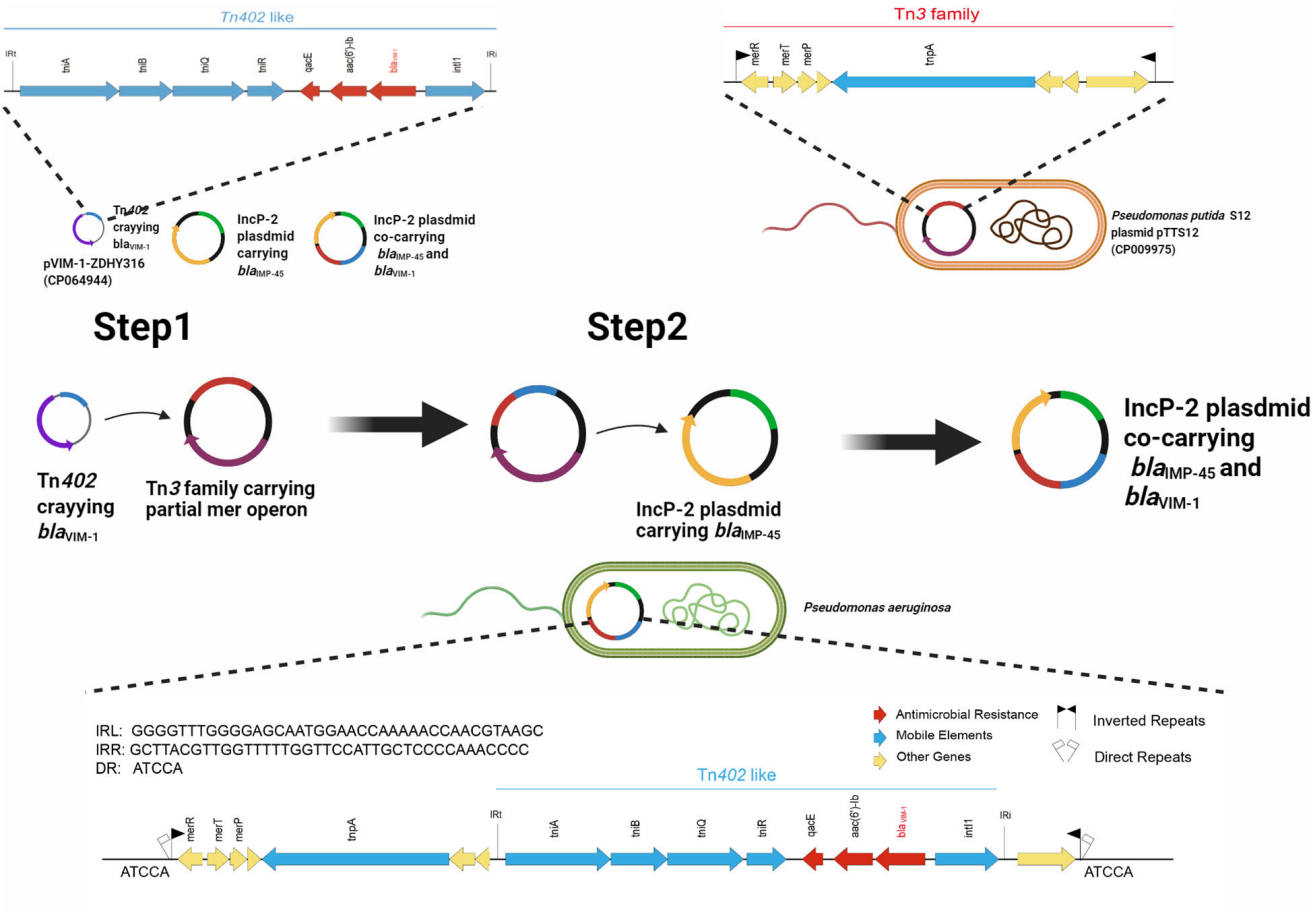
Evolutionary trajectory for the co-harboring *bla*_VIM-1_ and *bla*_IMP-45_ IncP-2 plasmids. Proposed formation mechanism of co-harboring *bla*_VIM-1_ and *bla*_IMP-45_ IncP-2 plasmids is as follows by sequence alignment. Step 1: Tn*402-*like transposon carrying *bla*_VIM-1_ was infused to Tn*3-*family transposon containing partial *mer* operon genes to generate a new Tn*3-*family transposon carrying *bla*_VIM-1_. Step 2: The new Tn*3-*family transposon mobilized onto the *bla*_IMP-45_-harboring IncP-2 plasmid, resulting in the emergence of the IncP-2 plasmid co-harboring both *bla*_VIM-1_ and *bla*_IMP-45_. **Figure** was created with BioRender.

Third-generation sequencing reveals that the genome of the CJ05 strain contains a chromosome and an IncP-2 megaplasmid (**Figure 2A**). This plasmid carries *bla*_VIM_, *bla*_IMP_, and other resistance genes like *tmexCD3-toprJ3*. The *bla*_VIM-1_ gene is located on a *Tn3* family transposon (**Figure 2C**), including a complete *Tn402* module. The genetic background of *bla*_IMP-45_ is similar to previously reported IncP-2 plasmids carrying *bla*_IMP-45_(**Supplementary Table S1**), situated on a Tn*6485-*like transposon (**Figures 2B** and **3**). The *bla*_IMP-45_ gene is found directly downstream of the transposable element Tn*As1* and within a class 1 integron, In*786*, with the gene arrangement *intI1-aacA4-blaIMP-45-blaOXA-1-catB3*. In*786* is located within a Tn*6485-*like transposon. Similar genetic contexts have been detected or reported in several other IncP-2 plasmids, including pPA166-2-MDR, pR31014-IMP, pNF143349, and pPAHT-1 (**Figure 2B**). Subsequent snp-based phylogenetic analysis of the *bla*_IMP-45_-carrying IncP-2 plasmid revealed that pCJ05 is homologous to other *bla*_IMP-45_-carrying IncP-2 plasmids (**Figure 3**). Based on this observation, we hypothesize that pCJ05 acquired *bla*_VIM-1_ through horizontal gene transfer (HGT) from a *bla*_IMP-45_-carrying IncP-2 plasmid. Further analysis of the genetic context surrounding *bla*_VIM-1_ revealed direct evidence of transposition. Specifically, we identified both direct repeat (DR) and inverted repeat (IR) sequences flanking *bla*_VIM-1_. We discovered that *bla*_VIM-1_ is carried by a Tn*402*-like transposon, which subsequently inserted into a Tn*3*-family transposon harboring partial *mer* operon genes, resulting in the formation of a novel Tn*3*-family transposon (**Figure 4**).The Tn*402*-like transposon was found to carry a complete tniABQR transposition module, while the Tn*3*-family transposon with partial *mer* operon contained *tnpA* but lacked *tnpR*. Comparative analysis using the NCBI database identified two plasmids carrying these transposons, pVIM-1-ZDHY316(CP064944) and pTTS12(CP009975), respectively. Based on these findings, we propose an evolutionary trajectory for the co-harboring *bla*_VIM-1_ and *bla*_IMP-45_ IncP-2 plasmid. First of all, the Tn*402*-like transposon carrying *bla*_VIM-1_ transposed into the Tn*3*-family transposon containing partial *mer* operon genes, forming a novel *bla*_VIM-1_-carrying Tn*3*-family transposon. This composite transposon was subsequently mobilized onto the *bla*_IMP-45_-harboring IncP-2 plasmid, ultimately resulting in the emergence of an IncP-2 plasmid co-harboring both *bla*_VIM-1_ and *bla*_IMP-45_ (**Figure 4**).

## Discussion

CRPA carrying carbapenemases is an opportunistic Gram-negative pathogen and a common cause of nosocomial infections, particularly respiratory tract infections. The IncP-2 plasmid harboring the *bla*_IMP-45_ gene was initially identified in China and subsequently disseminated rapidly across the country. In 2014, canine-derived *P. aeruginosa* carrying *bla*_IMP-45_ on IncP-2 plasmids was first reported in Beijing (Wang et al., 2014). Subsequently, the IncP-2 plasmids harboring *bla*_IMP-45_ was identified in *P. aeruginosa* isolates from various regions in China, spanning multiple sequence types (STs), including high-risk STs such as ST277 and ST463, indicating that *P. aeruginosa* with IncP-2 plasmids carrying *bla*_IMP-45_ has been disseminated in the country. IncP-2 plasmid subtypes facilitate the spread of *bla*_IMP-45_ among genetically diverse *P. aeruginosa* and have incorporated various resistance genes during their evolution, such as *bla*_AFM-1_, *bla*_PER-1_, *tmexCD-oprJ*, *armA*, and *qnrVC1 (* Zhang et al., 2021; Dong et al., 2022; Zhou et al., 2023). In 2018, the ST277 *P*. *aeruginosa* strain PA298 (CP040127), co-harboring *bla*_VIM-1_ and *bla*_IMP-45_, was identified in Guangzhou, China, with both resistance genes located on the IncP-2 plasmid pBM908 (CP040126). Later, in 2022, VIM-1–IMP-45-CRPA strains NF143349 (CP114761) and PAHT-1 (CP163544) were detected in Guangzhou, Zhuhai, and Shanxi, belonging to MLST types ST277 and ST188, respectively. Comparative analysis revealed that their plasmids shared 97% and 98% coverage, with 99.1% and 100% identity to pCJ05, respectively. A recent study also revealed that IncP-2 plasmids carrying *bla*_NDM-1_ and *bla*_VIM-2_ have caused neonatal sepsis in Morocco (Daaboul et al., 2024). IncP-2 plasmids are increasingly becoming reservoirs for metallo-β-lactamases in *Pseudomonas* spp worldwide.

In the study, we documented an outbreak of *P. aeruginosa* co-producing the VIM-1 and IMP-45 in the IncP-2 plasmid among transplant patients in a tertiary hospital in China. Our study documented the persistent clonal dissemination of VIM-1–IMP-45 CRPA strains in a tertiary hospital and investigates a possible evolutionary trajectory for the emergence of these strains. In our study, among 10 patients infected with the VIM-1-IMP-45-CRPA strain, two patients who did not receive CZA+AZT combination therapy developed bloodstream infections caused by the VIM-1-IMP-45-CRPA strains. This suggests that CZA+AZT combination therapy may reduce the risk of bloodstream infections caused by the VIM-1-IMP-45-CRPA strains in transplant patients. Our research also indicates that cefiderocol could be a better treatment option for VIM-1–IMP-45 CRPA strains. Furthermore, PBP3 has been shown to be a common adaptive target among *P. aeruginosa* isolates from patients treated with β-lactams (Clark et al., 2019). In addition, the mechanisms of aztreonam resistance in *P. aeruginosa* are diverse, including the overexpression of the *mexAB-oprM* efflux system, alterations in *ftsI* (PBP3) leading to disrupted drug binding, and mutations or overexpression of chromosomal *ampC* β-lactamase or changes in its coding sequence, which enhance active drug efflux. Previous studies (Jorth et al., 2017; Clark et al., 2019; McLean et al., 2019), as well as our research, have identified the PBP3 (P527S) mutation in clinical *P. aeruginosa* strains resistant to aztreonam. The P527S mutation is hypothesized to contribute to resistance, not conclusively demonstrated. Through molecular docking analysis, structural bioinformatics revealed that the PBP3 (P527S) mutation results in reduced binding affinity to aztreonam. However, whether this slight reduction in binding affinity is sufficient to cause a significant increase in aztreonam MIC values requires further investigation to confirm. To our knowledge, this is the first report of an outbreak of VIM-1–IMP-45 CRPA strains among transplant patients.

In summary, the VIM-1–IMP-45 CRPA strains exhibit extensive antimicrobial resistance and resulted in a high mortality rate, enabling them to withstand host defenses and clinical interventions in transplant patients, thereby facilitating their sustained transmission. Infections caused by these strains are associated with high mortality rates, particularly among immunocompromised transplant recipients, indicating the critical need for effective infection prevention and control strategies.

## Data Availability

The datasets presented in this study can be found in online repositories. The names of the repository/repositories and accession number(s) can be found below: https://www.ncbi.nlm.nih.gov/, BioProject: PRJNA1222284.
