# Supplementary material for: Tracing the evolutionary trajectory of the IncP-2 plasmid co-harboring bla IMP-45 and bla VIM-1: an outbreak of Pseudomonas aeruginosa co-producing IMP-45 and VIM-1 carbapenemases in China
Source: Front Cell Infect Microbiol. 2025 Jun 19;15:1623241. doi: 10.3389/fcimb.2025.1623241 (PMC12222221; doi:10.3389/fcimb.2025.1623241)
Supplement: Supplementary file 1 [file DataSheet1.docx]

Supplemental Table1:Previously reported IncP-2 plasmids carrying bla_IMP-45_^*^

| **Plasmid** | **S**train | **S**train | **Collection date** | | **Location**  **province/country** | **β-lactams** [**resistance gene**](#TF5) | **GenBank** accession number |
| --- | --- | --- | --- | --- | --- | --- | --- |
|  |  | **ST** |  |  |  |  |  |
| pBM413 | [*P. aeruginosa* PA121617](https://www.ncbi.nlm.nih.gov/nuccore/PA121617) | ST389 | | 2012 | Guangzhou, China | *bla*IMP-45, *bla*OXA-1 | CP016215 |
| pA681-IMP | *P. aeruginosa* | ST274 | | 2014 | China | *bla*IMP-45, *bla*OXA-1 | MF344570 |
| pPAG5 | *P. aeruginosa* PAG5 | unknown | | 2016 | China | *bla*IMP-45, *bla*OXA-1 | CP045003 |
| pHS17-127 | *P. aeruginosa* HS17-127 | ST369 | | 2017 | Shanghai, China | *bla*IMP-45, *bla*OXA-1, *bla*PER-1, *bla*AFM-1 | CP061377 |
| pBM908 | *P. aeruginosa* PA298 | ST277 | | 2018 | China | *bla*IMP-45, *bla*OXA-1 | CP040126 |
| pPA166-2-MDR | *P. aeruginosa* PA166-2 | ST313 | | 2019 | Shanxi China | *bla*IMP-45, *bla*OXA-1 | JAKHEW010000003 |
| pNY5087-IMP | *P. aeruginosa* NY5087 | unknown | | 2019 | Henan China | *bla*IMP-45, *bla*OXA-1 | CP132348 |
| pPA942-IMP45 | *P. aeruginosa* PA942 | ST1428 | | 2020 | Hangzhou China | *bla*IMP-45, *bla*OXA-1, *bla*PER-1 | CP129201 |
| pPA30_1 | *P. aeruginosa* PA30 | ST463 | | 2021 | Zhejiang China | *bla*IMP-45, *bla*OXA-1, *bla*PER-1, *bla*AFM-1 | CP104871 |
| pPAHT-1 | *P. aeruginosa* PAHT-1 | ST181 | | 2022 | China | *bla*IMP-45, *bla*OXA-1 | CP163545 |
| pNF143349 | *P. aeruginosa* NF143349 | ST277 | | 2022 | Guangzhou, China | *bla*IMP-45, *bla*OXA-1 | CP114762 |
| pPA64-1 | *P. aeruginosa* PA64 | ST463 | | 2022 | Zhejiang China | *bla*IMP-45, *bla*OXA-1 | CP159838 |
| pR31014-IMP | *P. aeruginosa* | unknown | | unknown | China | *bla*IMP-45, *bla*OXA-1 | MF344571 |
| p727-IMP | *P. aeruginosa* | unknown | | unknown | China | *bla*IMP-45, *bla*OXA-1 | MF344568 |
| pSY153-MDR | *P. putida* SY153 | unknown | | unknown | Hainan, China | *bla*IMP-45, *bla*OXA-1 | KY883660 |

^*^ ST, sequence type

Supplemental Table 2: Antimicrobial drug susceptibility profiles for the *Pseudomonas aeruginosa* strains AS01, AS02 and TC01

| Strains | MIC (μg/mL) | | | | | | | | | | | | | | |
| --- | --- | --- | --- | --- | --- | --- | --- | --- | --- | --- | --- | --- | --- | --- | --- |
|  | MEM | IMP | CAZ | TCC | FEP | AZT | AK | TOB | LEV | CIP | P/T | CZA | COL | SCF | FDC |
| AS01 | >16 | >16 | >64 | >128 | >32 | >32 | ≤2 | ≤1 | >8 | >4 | >128 /4 | 0.5/4 | 2 | >64 | 1 |
| AS02 | >16 | >16 | >64 | >128 | >32 | >32 | ≤2 | ≤1 | >8 | >4 | >128 /4 | >128/4 | 2 | >64 | >64 |
| TC01 | >16 | >16 | >64 | >128 | >32 | >32 | >64 | >16 | >8 | >4 | >128 /4 | >128/4 | 2 | >64 | >64 |
| ATCC 27853 | 0.5 | 2 | 2 | 16 | 2 | 2 | ≤2 | ≤1 | 1 | 0.5 | 1/4 | 0.5/4 | 2 | ≤8 | 0.125 |

^*^ AK, amikacin; ATCC, American Type Culture Collection (<https://www.atcc.org>); AZT, aztreonam; CAZ, ceftazidime; CIP, ciprofloxacin; COL, colistin; CRPA, carbapenem-resistant *Pseudomonas aeruginosa*; CZA, ceftazidime/avibactam;FDC, cefiderocol; FEP, cefepime; IPM, imipenem; VIM,Verona integron-encoded metallo-β-lactamase; LEV, levofloxacin; MEM, meropenem; NA, not applicable; P/T,piperacillin/tazobactam; ST313, sequence type 313;SCF,cefoperazone/sulbactam; TOB,tobramycin; TCC,ticarcillin/clavulanic acid

Supplemental Table 3: The detail of 46 ST313 *Pseudomonas aeruginosa* strains from NCBI database

| **Strain** | **GenBank assembly** | **Country** | **Collection time** | **Isolation type** |
| --- | --- | --- | --- | --- |
| 1098 | GCF_003840225.1 | United Kingdom | Unknown | Canis lupus |
| VRFPA07 | GCF_000506805.1 | India: Chennai | 2013 | Homo sapiens |
| PA11 | GCF_028331495.1 | Thailand: Songkhla | 2017 | Homo sapiens |
| PA166-2 | GCF_025370235.1 | China: Shanxi | 2019 | Chicken |
| 138 | GCF_004350215.1 | Italy: Trento | 2018 | Homo sapiens |
| 14-0035 | GCF_028435485.1 | Spain: Santander | 2012 | Homo sapiens |
| 159-2 | GCF_030326425.1 | China | 2013 | Chicken |
| 164-1 | GCF_030326405.1 | China | 2019 | Chicken |
| 166-2 | GCF_030326365.1 | China | 2019 | Chicken |
| 2022CK-00068 | GCF_029962165.1 | USA | 2022 | Homo sapiens |
| 4018_S3 | GCF_021060605.1 | Spain | 2019 | Homo sapiens |
| AUS517 | GCF_003840825.1 | Australia: Brisbane | 2007 | Unknown |
| AZPAE14359 | GCF_000795785.1 | China: Shatin | 2008 | Unknown |
| AZPAE14393 | GCF_000796005.1 | Spain: Madrid | 2011 | Unknown |
| AZPAE14892 | GCF_000790885.1 | France: Besancon | 2008 | Unknown |
| AZPAE14932 | GCF_000793685.1 | Germany: Aachen | 2009 | Unknown |
| AZPAE15059 | GCF_000793245.1 | France: Nantes | 2010 | Unknown |
| BL25 | GCF_000480645.1 | USA | Unknown | Unknown |
| C1.3 | GCF_026727735.1 | Germany: Bielefeld | 2019 | Plastic surface of detergent compartment of a washing machine |
| C2-144 | GCF_013169135.1 | Colombia:Antioquia, Bello | 2015 | Homo sapiens |
| Cotonu1 | GCF_003836245.1 | Benin: Cotonu | Unknown | River water |
| CPa40 | GCF_011682995.1 | China: Beijing | 2017 | Homo sapiens |
| ENV-245 | GCF_003633335.1 | Estonia | 2014 | Sewage |
| FBPa_K5993 | GCF_030069145.1 | Netherlands: Utrecht | 2019 | Homo sapiens |
| FBPa_L1150 | GCF_030068955.1 | Netherlands: Utrecht | 2019 | Homo sapiens |
| GD03837 | GCF_029841325.1 | Pakistan | 2019 | Unknown |
| GD04090 | GCF_029836195.1 | Pakistan | 2018 | Unknown |
| GD04128 | GCF_029835495.1 | Pakistan | 2018 | Unknown |
| HUM-227 | GCF_003633285.1 | Estonia | 2012 | Homo sapiens |
| Jp222 | GCF_003975325.1 | Japan: Pacific Ocean | 2004 | Sea water |
| K19PSE24 | GCF_013201015.1 | South Korea: Busan | 2017 | Unknown |
| NSPa40 | GCF_011683035.1 | China: Beijing | 2018 | Homo sapiens |
| Ocean-222 | GCF_002263575.1 | Pacific Ocean | 2004 | Open Ocean |
| PAL1.61 | GCF_013912485.1 | France: Lille | Unknown | Homo sapiens |
| PAL1.63 | GCF_013912425.1 | France: Lille | Unknown | Homo sapiens |
| PaLo514 | GCF_027855735.1 | Switzerland | 2012 | Homo sapiens |
| Ps200 | GCF_025698595.1 | Saudi Arabia: Riyadh | Unknown | Homo sapiens |
| S13 | GCF_013177105.1 | China:Taiwan | 2006 | Homo sapiens |
| SC34 | GCF_029955105.1 | China: Sichuan | 2018 | Homo sapiens |
| VET-27 | GCF_003633295.1 | Estonia | 2012 | Dog |
| W15Apr4 | GCF_003975285.1 | Belgium: Woluwe river | 2002 | River |
| WH-SGI-V-07255 | GCF_001453835.1 | USA:Boston | 1997 | Unknown |
| WH-SGI-V-07258 | GCF_001452935.1 | Belgium | 1998 | Unknown |
| WH-SGI-V-07636 | GCF_001452155.1 | USA | 2005 | Unknown |
| BWH050 | GCF_000629585.1 | Unknown | 2015 | Unknown |
| R3890 | GCF_003954745.1 | Unknown | 2012 | Unknown |

Supplemental Figure 1: The SNPs matrix of 17 ST313 VIM-1–IMP-45–CRPA strains.


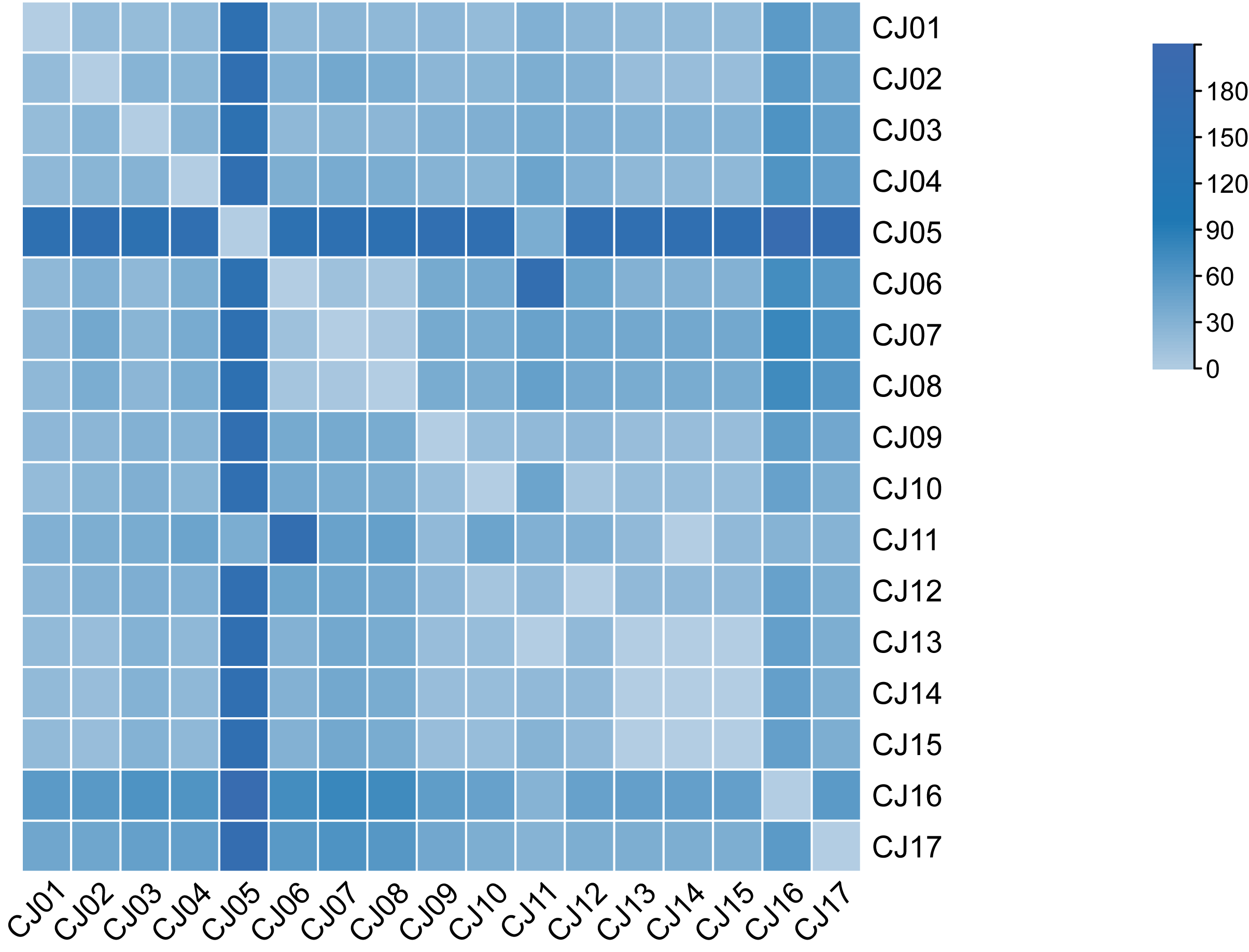


Supplemental Figure 2: The main virulence genes of 17 ST313 VIM-1–IMP-45–CRPA strains.


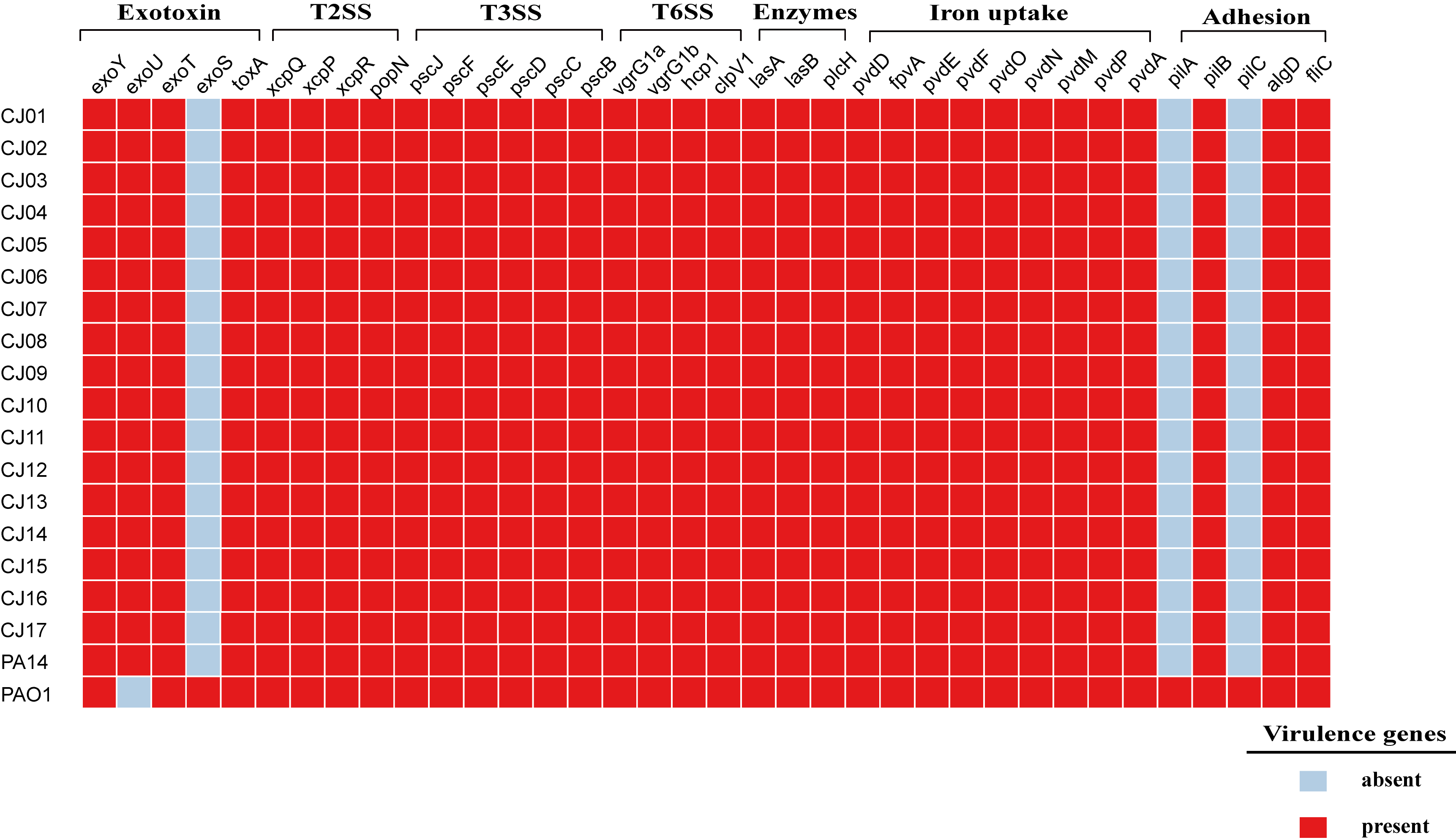


Supplemental Figure 3:Phylogenetic analysis of plasmids of VIM-1–IMP-45–CRPA strains.^*^

^
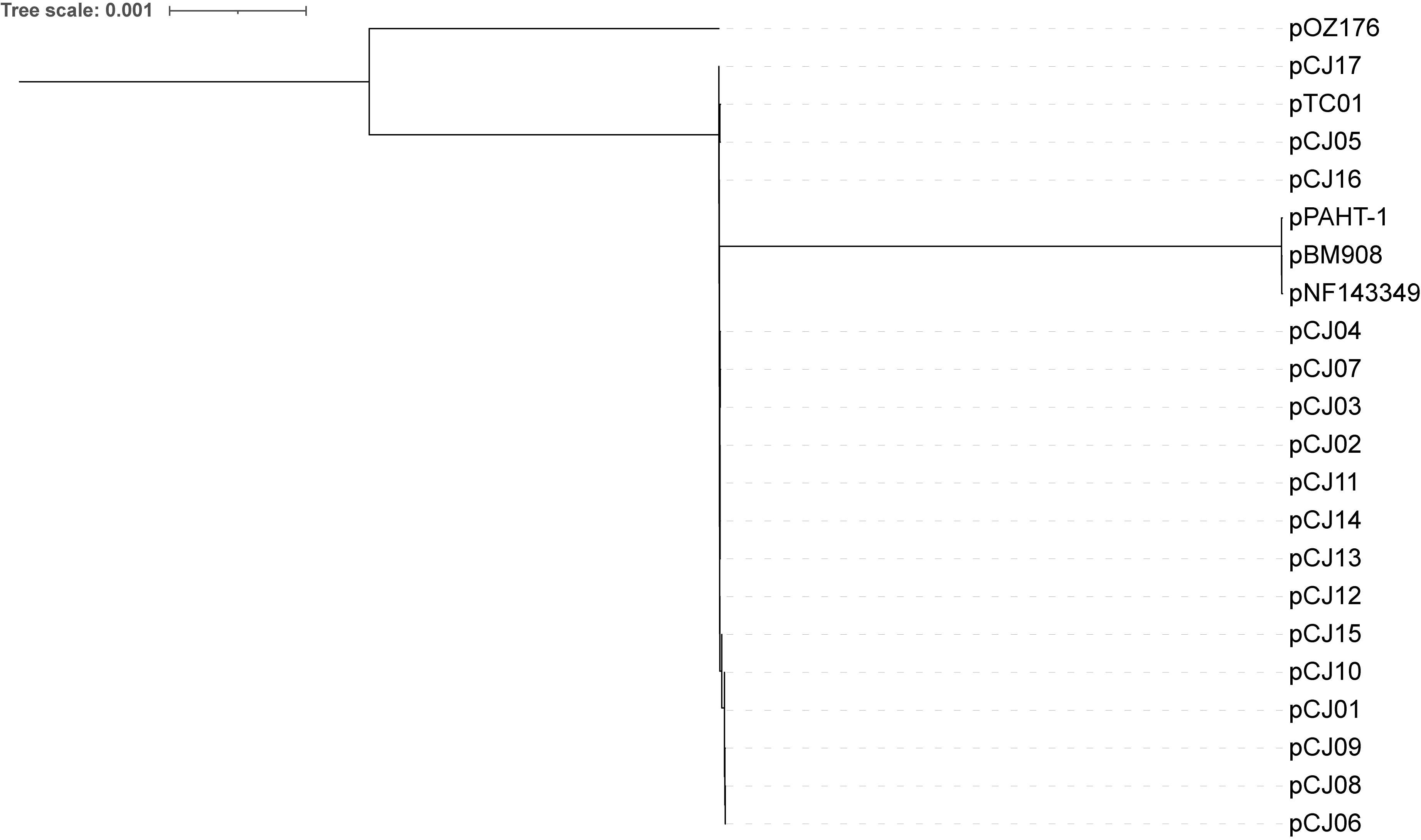
^

^*^The IncP-2 plasmid pOZ176 was used as a reference.

Supplemental Figure 4: Binding analysis of aztreonam with PBP3(A) and PBP3(P527S) (B).


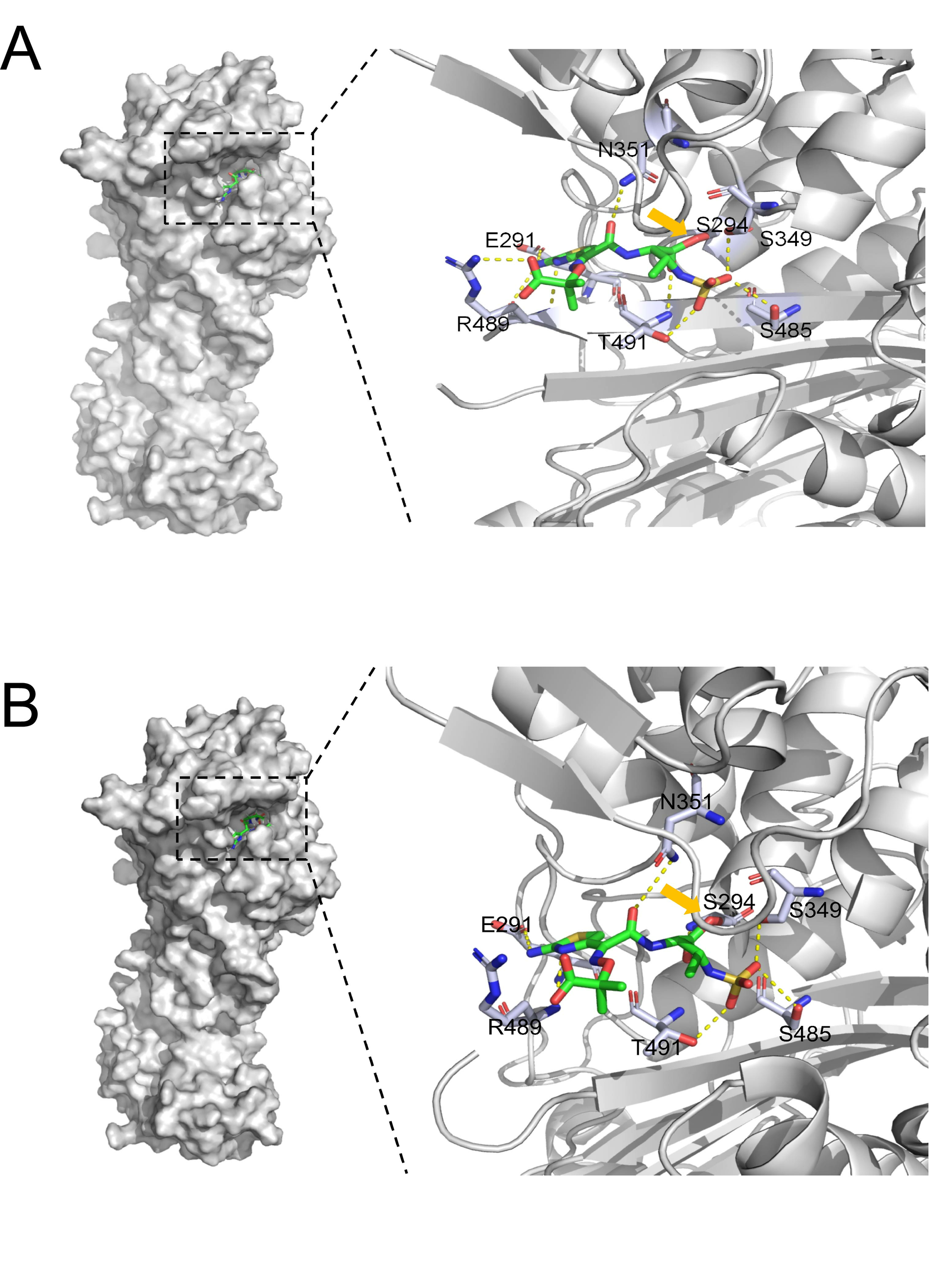


The best-fitted molecular docking calculated using the software Schrödinger.

The binding region between PBP3 and aztreonam is marked in a rectangle. The amide bond formed by aztreonam with Ser (294) is marked with a orange arrow and the hydrogen bond formed by aztreonam with PBP3 is indicated by a yellow dashed line. Structure figures are visualized using PyMol.
